# Supplementary material for: Duchenne muscular dystrophy cell culture models created by CRISPR/Cas9 gene editing and their application in drug screening
Source: Sci Rep. 2021 Sep 14;11:18188. doi: 10.1038/s41598-021-97730-5 (PMC8440673; doi:10.1038/s41598-021-97730-5)
Supplement: Supplementary file 1 — Supplementary Information. [file 41598_2021_97730_MOESM1_ESM.pdf]

DUCHENNE MUSCULAR DYSTROPHY CELL CULTURE MODELS CREATED BY CRISPR/Cas 9 GENE EDITING AND THEIR APPLICATION IN DRUG SCREENING

P. Soblechero-Martín[1,2], E. Albiasu-Arteta[1], A. Anton-Martinez[1], L. de la Puente-Ovejero[1], I. Garcia-Jimenez[1], G. González-Iglesias[1], I. Larrañaga-Aiestaran[1], A. López-Martinez[1], J. Poyatos-García[3], E. Ruiz-Del-Yerro[1], F. Gonzalez[4], V. Arechavala-Gomeza[1,5]

[1]Neuromuscular Disorders, Biocruces Bizkaia Health Research Institute, Barakaldo, Spain
[2]Osakidetza Basque Health Service, Bilbao-Basurto Integrated Health Organisation, Basurto University Hospital, Clinical Laboratory Service, Bilbao, Spain
[3]La Fe Health Research Institute, Hospital La Fe, Valencia, Spain
[4]Pluripotent Stem Cells and Activation of Endogenous Tissue Programs for Organ Regeneration (PR Lab), Institute for Bioengineering of Catalonia (IBEC), Barcelona, Spain
[5]Ikerbasque, Basque Foundation for Science, Bilbao, Spain

SUPPLEMENTARY FILES

Supplementary table 1. Gene edition and antisense experiments primer sets.

NAME	PRIMER SEQUENCE	APLICATION	TARGET	AMPLICON LENGTH (bp) (NON EDITED OR NON SKIPPED)	AMPLICON LENGTH (bp) (EDITED OR SKIPPED)
**DMD-Seq-D52-DOWN-F2**	TTTCTAAAAGTGTTTTGGCTGGTC	Editing confirmation	DNA	750	228
**DMD-Seq-D52-DOWN-R2**	TACCAAAGTTCCTGCCCACC	Editing confirmation	DNA	750	228
**UTRN F1**	TGATGGTACCTCCACCTACATCT	Editing confirmation	DNA	692	421
**UTRN R1**	TTACTTCCCATTGTTACTGCAA	Editing confirmation	DNA	692	421
**47F**	AGTGCTCCCATAAGCCCAGAA	Skipping confirmation	RNA	1265	1147
**54R**	GAAGTTTCAGGGCCAAGTCA	Skipping confirmation	RNA	1147	914
**49F**	CCAGCCACTCAGCCAGTG	Skipping confirmation	RNA	1147	914
**53R**	TTGCCTCCGGTTCTGAAGG	Skipping confirmation	RNA	422	189

Supplementary table 2. Off-target primer sets.

**Primers off targets (5'—3'):**	
**Ob1_2_Off1_F**	ATGCTTCTCATTTGCTGCCTGATG
**Ob1_2_Off1_R**	GCTGTCACGATCTGATTGGAGTTTC
**Ob1_2_Off1_Seq**	TCTCCAGGCTCCAAGTAT
**Ob1_2_Off2_F**	ACAAGAAAGTCCTGGGTGTCA
**Ob1_2_Off2_R**	GACTTGTCAGCCTCCATAATCATT
**Ob1_2_Off2_Seq**	CTGATGAGGGCTTTCCAG
**Ob1_2_Off3_F**	AATGGCAGTTTTGGGAAATTCA
**Ob1_2_Off3_R**	CATTTTTTGTTGTCTGTGCCC
**Ob1_2_Off3_Seq**	TAAGTTACAATGCTTCCTGAA
**Ob1_2_Off4_F**	TACTTAATCCCCGTGTGTCTCAGT
**Ob1_2_Off4_R**	ATGCAATGTGAAGGCTGTCCG
**Ob1_2_Off4_Seq**	TTCCTTGTTTGTGCCTCA
**Ob1_2_Off5_F**	TCAAGAAAGTATGGTGTTGGTGAA
**Ob1_2_Off5_R**	AATTGTGTGAGTCCATTCCCATAA
**Ob1_2_Off5_Seq**	TTGACAAAGGGGCAAAGG
**Ob1_2_Off6_F**	ATGGCAAATAGTTACAATGTCA
**Ob1_2_Off6_R**	AATTTACACAACTAGAGCCATCT
**Ob1_2_Off6_Seq**	ACAGGAAGTCTTGAAAAAAGTA
**Ob1_6_Off1_F**	TTCCCTTGAACTTGCGGAGGT
**Ob1_6_Off1_R**	CCTTTTGGTTCCTGTGCCCC
**Ob1_6_Off1_Seq**	GTGCTCAAGCTCCCTTAT
**Ob1_6_Off2_F**	GCAGCAGATGGGTTAGGAGG
**Ob1_6_Off2_Seq**	CATGTAGGAATGACAGGAGT
**Ob1_6_Off2_R**	CTTGTGGCCAGCATCAGGTA
**Ob1_6_Off3_F**	CTGCTAGAGTGAGAGAACTGTGG
**Ob1_6_Off3_R**	GGAAACCAGGGCAAATCATGTCT
**Ob1_6_Off3_Seq**	GCCCCACATAGGACAAAT
**Ob1_6_Off4_F**	GCTGAAGGAAGTTCCAGGCA
**Ob1_6_Off4_R**	CAGGCTGGCAAGATGGAGAA
**Ob1_6_Off4_Seq**	GGCATCCTTATAGCAATTTTT
**Ob1_6_Off5_F**	GATAGCCCCACCAGACAACC
**Ob1_6_Off5_R**	AGGGCCAAATCCTCACAACC
**Ob1_6_Off5_Seq**	TCACCATTCTCATCCCCT
**Ob1_6_Off6_F**	TCAGAAAGGCTTGCCCTCA
**Ob1_6_Off6_R**	CTCACATGGCAAGTGGGGAT
**Ob1_6_Off6_Seq**	CCATCTAAAATCACCACACC
**Ob2_22_Off1_F**	ATGAGCCTCACAGATGCCTG
**Ob2_22_Off1_R**	GAAGACAGGGCCTGGATGTC
**Ob2_22_Off1_Seq**	TTAAAGTCTGTGCCCCTC
**Ob2_22_Off2_F**	AGGCTCTGCAGTTCAACCTC
**Ob2_22_Off2_R**	AACAGGCTCCAAACGTGTGA
**Ob2_22_Off2_Seq**	AATGACTTATACAGGGGACAT
**Ob2_22_Off3_F**	GGCCTTCTTTCGTGGACAGA

**Ob2_22_Off3_R**	GAATCATAGGCCTCCCGTGG
**Ob2_22_Off3_Seq**	TCGTGTTCTCTGTTGTGA
**Ob2_22_Off4_F**	GCCATAATCACACATCAAACCCT
**Ob2_22_Off4_R**	TGTTGCCATGCGAATTCGAG
**Ob2_22_Off4_Seq**	GAGAAGTTGAGGGAACCG
**Ob2_22_Off5_F**	GAGGTGGCATTCGGTAAAAGTTC
**Ob2_22_Off5_R**	TTGACAACCACGGGAGGCAG
**Ob2_22_Off5_Seq**	TGATTCTTTCCAGGCTCAT
**Ob2_22_Off6_F**	GGAGTGTGAGGGCTTCCTTC
**Ob2_22_Off6_R**	AGATGCCTGCTTACCTGCTG
**Ob2_22_Off6_Seq**	CATCTGTGTTCTCTGTTGTG
**Ob2_26_Off1_F**	GGGCAGGCTTGGGAGACATA
**Ob2_26_Off1_R**	GTGTCCAGCCCATTCTTTGAAGT
**Ob2_26_Off1_Seq**	TGCTCCCACTGCTGTTAG
**Ob2_26_Off2_F**	AGGTGCTCGCTTCTTTCCAA
**Ob2_26_Off2_R**	CCAGAAGTGAAGCTTTGCACC
**Ob2_26_Off2_Seq**	AACTTCTTGCACAGCCTT
**Ob2_26_Off3_F**	GGCATTCCTAGATCAGTGTGTGC
**Ob2_26_Off3_R**	CCCCAACTCAAACCAAGACGG
**Ob2_26_Off3_Seq**	CTGGGGGGATGTTACTGT
**Ob2_26_Off4_F**	TGGCTCTGTTTCTTGCCCAA
**Ob2_26_Off4_R**	TGGTTACTGGGCAGACATGG
**Ob2_26_Off4_Seq**	AAGCTAAAAGACATTGACAGT
**Ob2_26_Off5_F**	CACTGGAAAGAACATGGGCTCTG
**Ob2_26_Off5_R**	TGGTGTGTCCTGGGAGCATC
**Ob2_26_Off5_Seq**	CTCGGTTTCTACTGTGTGA
**Ob2_26_Off6_F**	TTTTATGTCCCCCACCCCTCA
**Ob2_26_Off6_R**	CCATGCCCAGCCCTAGTTTG
**Ob2_26_Off6_Seq**	GGGAAGCAGAGAAGTTGT

SUPPLEMENTARY FIGURES

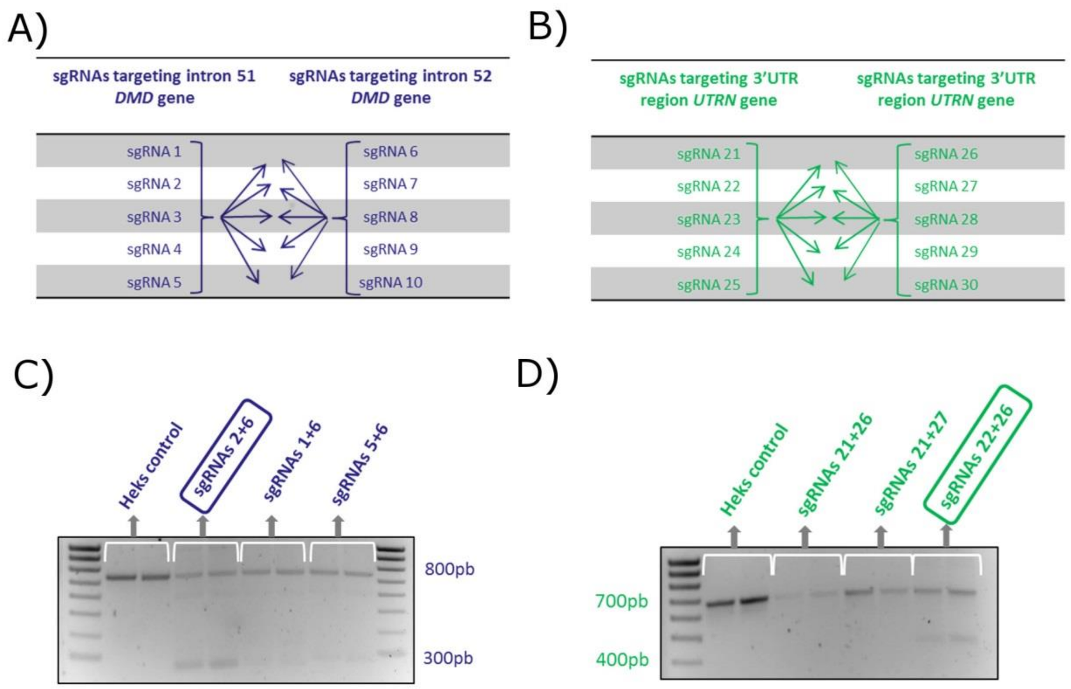

Supplementary figure 1. sgRNAs pairs test in HEK293 cells.

(A and B). Representation of all the different sgRNAs combinations tested for editing the *DMD* (A) and the *UTRN* loci (B). (C and D) Representative PCR analysis of HEK293 cells transfected with some of the sgRNAs combinations tested. Upper bands correspond to wild type or non-edited cells, while the lower bands correspond to the edited ones. Samples were analysed in duplicates (marked in white). Selected sgRNAs combinations are highlighted: Obj1sgRNA2 (C); Obj2 sgRNA22+sgRNA26 (D).

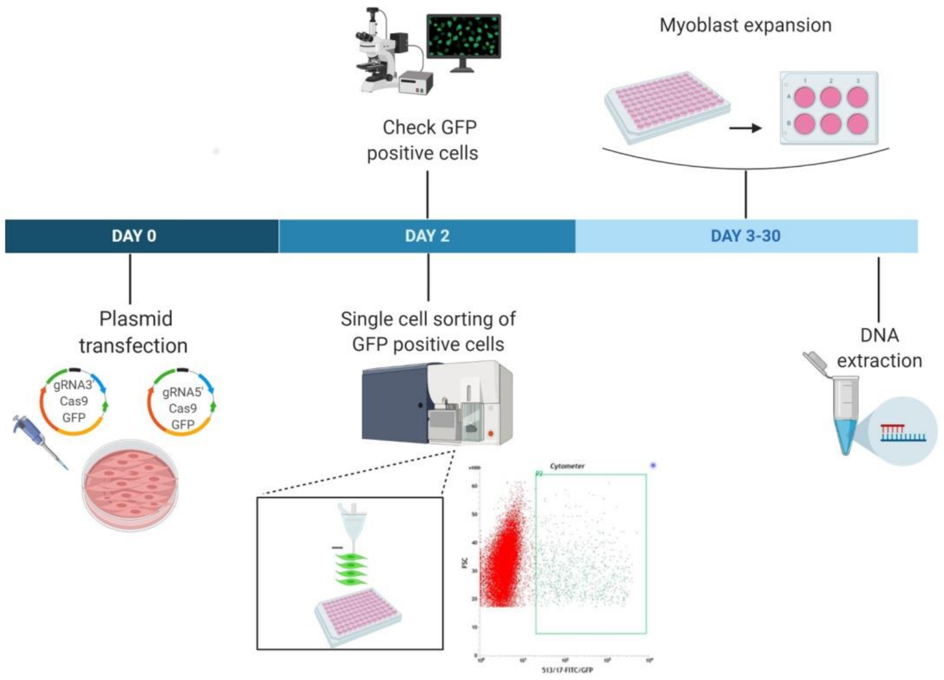

Supplementary figure 2. Cloning and editing workflow diagram.

Scheme of the workflow followed to obtain the edited myoblast cell lines. 48 hours after plasmid transfection GFP positive myoblasts were single cell sorted using FACS. Clones were expanded until confluence for DNA extraction. The dot plot shows GFP positive myoblasts (3,22 %) selected using FACS 48h post transfection with ViaFect™ reagent. Created with BioRender.com

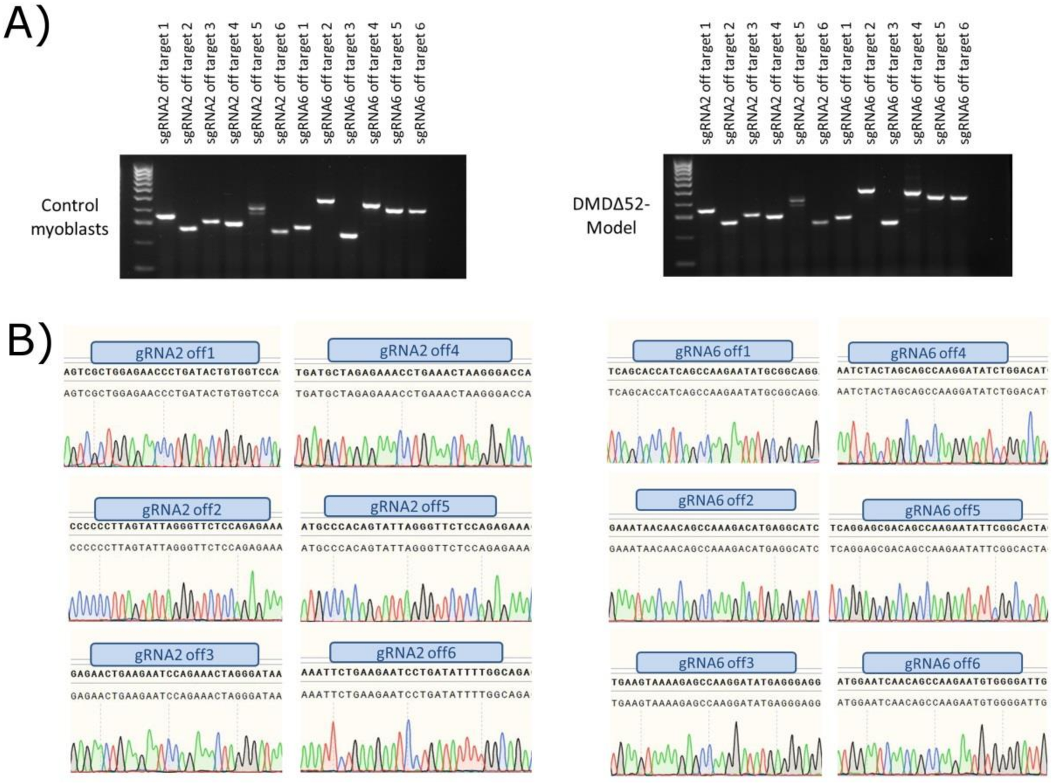

Supplementary figure 3. PCR Analysis of Off-target Effects. Representative gel and sequence of PCR analysis performed for all targets.

(A) Agarose gel showing the amplification of the six predicted off-targets regions for sgRNA2 and the six for sgRNA6 (the combination used for our DMD edition model) amplified in control myoblasts and DMDΔ52-Model. (B) All the amplicons were sequenced and no differences between control myoblasts and DMDΔ52-Model were found.

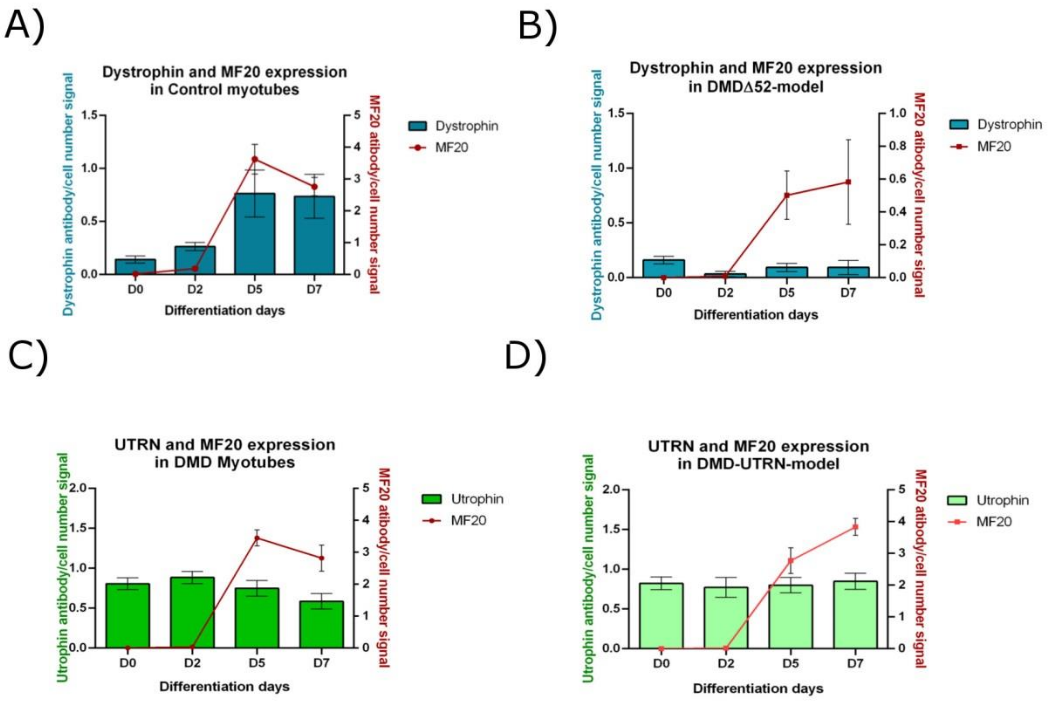

Supplementary figure 4. Myoblot assays at different fusion times in edited cells.

(A and B) Dystrophin and MF20 expression determined by myoblot in DMDΔ52-Model compared to control myoblasts along differentiation process. (C and D) Utrophin and MF20 expression determined by myoblot in DMD-UTRN-Model compared to DMD myoblasts. Myoblot analysis was performed using n=24 replicate wells for dystrophin or utrophin staining and n=18 replicate wells for MF20 staining.

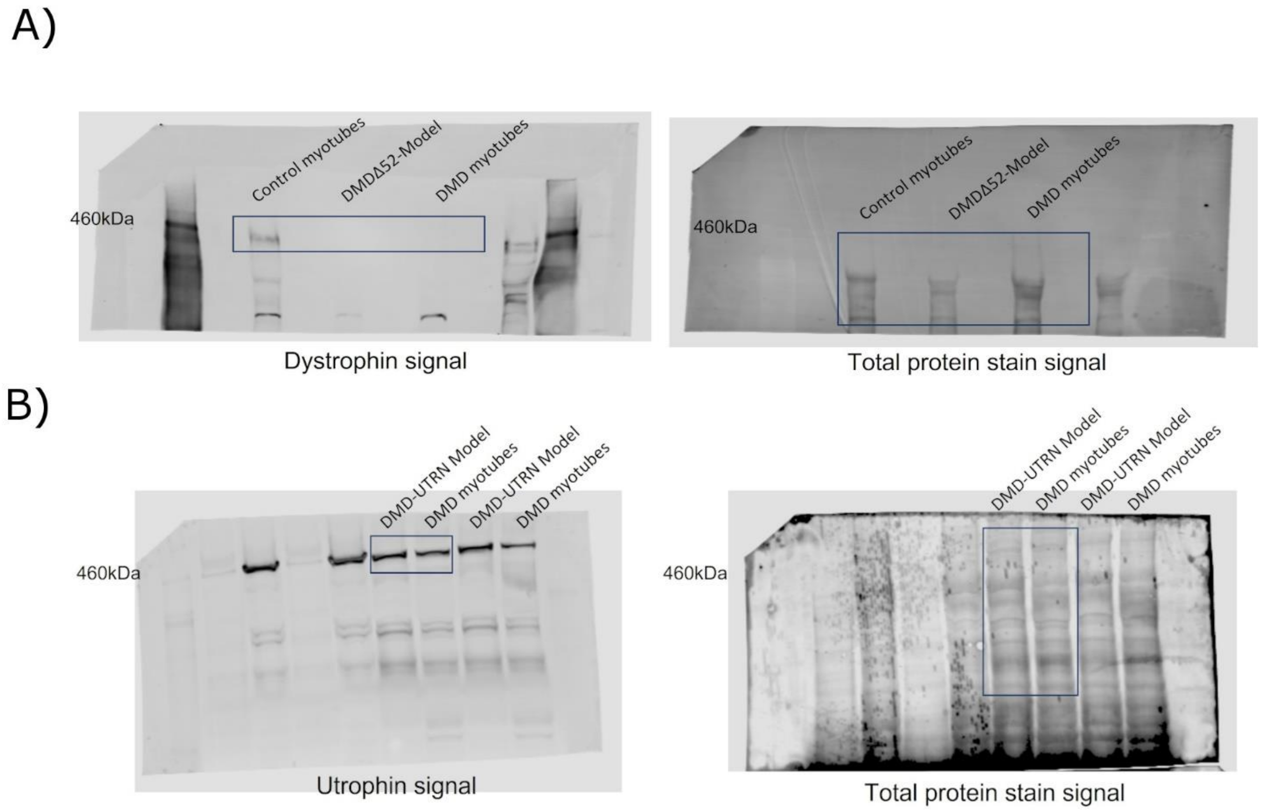

Supplementary figure 5. Full-length western blots.

(A) Full-length blots corresponding to figure 2B. Dystrophin signal is showed in the left blot and total protein stain signal is showed at the right. Cropped blot used for figure 2B is marked with a blue rectangle. (B) Full-length blots corresponding to figure 3B. Dystrophin signal is showed in the left blot and total protein stain signal is showed at the right. Cropped blot used for figure 3B is marked with a blue rectangle.
